# Severe COVID-19 Is Characterized by an Impaired Type I Interferon Response and Elevated Levels of Arginase Producing Granulocytic Myeloid Derived Suppressor Cells

**DOI:** 10.3389/fimmu.2021.695972

**Published:** 2021-07-14

**Authors:** Matthew J. Dean, Juan B. Ochoa, Maria Dulfary Sanchez-Pino, Jovanny Zabaleta, Jone Garai, Luis Del Valle, Dorota Wyczechowska, Lyndsey Buckner Baiamonte, Phaethon Philbrook, Rinku Majumder, Richard S. Vander Heide, Logan Dunkenberger, Ramesh Puttalingaiah Thylur, Bobby Nossaman, W. Mark Roberts, Andrew G. Chapple, Jiande Wu, Chindo Hicks, Jack Collins, Brian Luke, Randall Johnson, Hari K. Koul, Chris A. Rees, Claudia R. Morris, Julia Garcia-Diaz, Augusto C. Ochoa

**Affiliations:** ^1^ Louisiana State University Cancer Center, New Orleans, LA, United States; ^2^ Department of Surgery, Ochsner Medical Center, New Orleans, LA, United States; ^3^ Department of Genetics, LSU Health, New Orleans, LA, United States; ^4^ Department of Pediatrics, LSU Health, New Orleans, LA, United States; ^5^ Department of Pathology LSU Health, New Orleans, LA, United States; ^6^ Tissue Biorepository, Ochsner Medical Center, New Orleans, LA, United States; ^7^ Department of Biochemistry, LSU Health, New Orleans, LA, United States; ^8^ Department of Internal Medicine, Ochsner Medical Center, New Orleans, LA, United States; ^9^ School of Public Health, LSU Health, New Orleans, LA, United States; ^10^ Advanced Biomedical Computational Science, Frederick National Laboratory for Cancer Research, Frederick, MD, United States; ^11^ Division of Emergency Medicine, Boston Children’s Hospital and Department of Pediatrics, Harvard Medical School, Boston, MA, United States; ^12^ Department of Pediatrics, Emory University School of Medicine, Atlanta, GA, Children’s Healthcare of Atlanta, Atlanta, GA, United States

**Keywords:** COVID-19, coronavirus, G-MDSC, arginase, arginine, lung injury, interferon

## Abstract

COVID-19 ranges from asymptomatic in 35% of cases to severe in 20% of patients. Differences in the type and degree of inflammation appear to determine the severity of the disease. Recent reports show an increase in circulating monocytic-myeloid-derived suppressor cells (M-MDSC) in severe COVID 19 that deplete arginine but are not associated with respiratory complications. Our data shows that differences in the type, function and transcriptome of granulocytic-MDSC (G-MDSC) may in part explain the severity COVID-19, in particular the association with pulmonary complications. Large infiltrates by Arginase 1^+^ G-MDSC (Arg^+^G-MDSC), expressing NOX-1 and NOX-2 (important for production of reactive oxygen species) were found in the lungs of patients who died from COVID-19 complications. Increased circulating Arg^+^G-MDSC depleted arginine, which impaired T cell receptor and endothelial cell function. Transcriptomic signatures of G-MDSC from patients with different stages of COVID-19, revealed that asymptomatic patients had increased expression of pathways and genes associated with type I interferon (IFN), while patients with severe COVID-19 had increased expression of genes associated with arginase production, and granulocyte degranulation and function. These results suggest that asymptomatic patients develop a protective type I IFN response, while patients with severe COVID-19 have an increased inflammatory response that depletes arginine, impairs T cell and endothelial cell function, and causes extensive pulmonary damage. Therefore, inhibition of arginase-1 and/or replenishment of arginine may be important in preventing/treating severe COVID-19.

## Introduction

COVID-19 disease ranges from asymptomatic (35% of cases) to severe requiring treatment in intensive care units (https://www.cdc.gov/coronavirus/2019-ncov/hcp/clinical-guidance-management-patients.html). Approximately 20% of hospitalized COVID-19 patients develop hypoxemia, respiratory difficulty, hypercoagulation and end organ damage that may result in death. These clinical manifestations are similar to other coronavirus infections including SARS-CoV-1 and MERS-CoV. The pathophysiology is primarily associated with an over-active inflammatory response manifested by a cytokine release syndrome, a surge in granulocytes and decreased lymphocytes (1, 2). The mechanism(s) that regulate these events are unclear. Genomic studies of peripheral blood suggested an increase in genomic signatures associated with neutrophil functions in COVID-19 patients (3). Early reports also showed that increased neutrophils/lymphocyte ratios were associated with poor outcomes (4, 5). More recently, Agrati et al. (6), Reizine et al. (7) and Falck-Jones et al. (8) showed increased circulating monocytic - myeloid derived suppressor cells (M-MDSC) able to deplete arginine and inhibit T cell proliferation. MDSC have been best studied in cancer, but also described in chronic infections, autoimmunity, asthma and trauma (9–11). MDSC express high levels of arginase 1 (Arg1) that metabolizes arginine to ornithine and urea, effectively depleting this amino acid from the microenvironment. Rees et al (12) have reported alterations in amino acid levels, including arginine and ornithine, in COVID-19 patients. Another recent paper has described increased RNA expression of Arg1 in the peripheral blood mononuclear cells of COVID-19 patients, although they did not identify the cell of origin of this Arg1 (13). Arginine depletion inhibits T cell receptor signaling resulting in T cell dysfunction (14, 15). It also impairs nitric oxide production and increases endothelial cell dysfunction promoting intravascular coagulation (16, 17). Arginine depletion also increases the production of reactive oxygen species (ROS) which can damage infiltrated organs and exacerbate inflammation (18). We compared the type, function and transcriptomic signature of MDSC in COVID-19 patients to better understand their effect on arginine levels, T cell function and respiratory complications. Our results showed a major increase in circulating granulocytic-MDSC expressing high levels of arginase-1 (Arg^+^G-MDSC). Large accumulations of Arg^+^G-MDSC expressing NOX2 infiltrated the lungs of patients who died from severe COVID-19 complications. High numbers of Arg^+^G-MDSC in circulation depleted arginine in plasma, decreased T cell receptor zeta chain (CD3ζ) and increased markers of endothelial cell dysfunction. RNAseq studies demonstrated contrasting transcriptomes, where G-MDSC from asymptomatic COVID-19 patients had increased expression of type I IFN genes and pathways, while G-MDSC from severe COVID-19 patients had instead increased expression of genes associated with granulocyte functions and degranulation. These data support the concept that Arg^+^G-MDSC may play a significant role in the pathophysiology of COVID-19.

## Methods

### Patient Selection

The study was conducted under LSU IRB protocol #20-053 and Ochsner Medical Center IRB protocol 21015-101C. All participants were consented prior to inclusion in the study. The study included 24 severe, 5 asymptomatic, 26 convalescent COVID-19 patients and 15 healthy controls. All patients tested positive for SARS-CoV-2 by PCR. Severe patients were hospitalized while asymptomatic and convalescent patients remained outpatient. Healthy controls were negative either by PCR or for antibodies to SARS-Cov-2. Classification of the severity of COVID-19 was done using CDC criteria. All severe patients in this study were hospitalized in the intensive care unit for treatment because of hypoxia and respiratory distress or complications from pre-existing comorbidities. Autopsy samples from ten (10) patients who died from COVID-19 complications were collected at the LSU Health Science Center Pathology Department.

### Sample Processing

All sample processing was carried out in a BSL2 approved laboratory at LSU. Samples were fixed in formalin or lysed using standard lysis buffers or TRIzol prior to being removed from this laboratory. EDTA and sodium citrate anticoagulated peripheral blood samples were centrifuged for separation of plasma and cellular components. Plasma was frozen at -80°C and buffy coat was overlaid on ficoll-hypaque for separation of peripheral blood mononuclear cells (PBMC).

### Flow Cytometry

Briefly, whole blood or PBMC were stained for CD45 to identify immune cells, and antibodies against T cells (CD3, CD4 and CD8), NK cells (CD16), monocytes (CD68) and MDSC subpopulations. The latter were divided into G-MDSC (CD11b^+^CD66b^+^CD14^-^), and monocytic or M-MDSC (CD11b^+^ CD14^+^HLA-DR^low/neg^).

### Histology and Immunohistochemistry

Immunohistochemistry was performed using the avidin-biotin-peroxidase methodology, according to the manufacturer’s instructions (Vectastain ABC Elite Kit, Vector Laboratories, Burlingame, CA). Our modified protocol includes deparaffination in xylenes, rehydration through descending grades of ethanol up to water, non-enzymatic antigen retrieval with 0.01 M sodium citrate buffer pH 6.0 heated to 95°C for 25 minutes in a vacuum oven, endogenous peroxidase quenching with 3% H2O2 in methanol, blocking for 2 hours with normal horse serum (for mouse monoclonal antibodies) or normal goat serum (for rabbit polyclonal or recombinant rabbit monoclonal antibodies) and incubation with primary antibodies overnight at room temperature in a humidified chamber. After rinsing in PBS, sections were incubated with biotinylated secondary antibodies for 1 hour at room temperature, followed by incubation with avidin-biotin-peroxidase complexes for 1 hour. Finally, the peroxidase was developed with diaminobenzidine (Boehringer, Mannheim, Germany) for 3 minutes, and the sections were counterstained with Hematoxylin and mounted with Permount (Fisher Scientific, Pittsburgh PA). Antibodies used in this study for the characterization of immune cells included: a CD3 mouse monoclonal (Clone F7.2.38, 1:100 dilution, DAKO-Agilent Technologies, Santa Clara, CA), a CD20 mouse monoclonal Clone L26, 1:100 dilution, DAKO), a CD11b rabbit monoclonal, raised against the C-terminal (Clone EP1345Y, 1:100 dilution, Abcam, Cambridge, MA), a CD66b mouse monoclonal (Clone 80H3, 1:100 dilution, LifeSpan Biosciences, Seattle, WA), and a CD68 mouse monoclonal (Clone PG-M1, 1:100 dilution, DAKO). Other antibodies included a rabbit polyclonal anti-Arginase-1 (H-52, 1:500 dilution, Santa Cruz Biotechnology, Dallas, TX), a rabbit polyclonal anti-Nox1 (ab78016, 1:500 dilution, Abcam), and a mouse monoclonal anti-NOX2/gp91phox (Clone 54.1, 1:200 dilution, Abcam). Bright field photomicrographs were taken with an Olympus DP72 Digital Camera using an Olympus BX70 microscope (Olympus, Center Valley, PA).

### Double Labeling Immunofluorescence

Deparaffination and rehydration of tissues were performed as described above. Antigen retrieval was also performed with heat and citrate buffer; however, no endogenous peroxidase quenching is necessary. After overnight incubation with the first primary antibody (mouse or rabbit), sections were rinsed with PBS, and an AlexaFluor-568-tagged secondary antibody was incubated for 2 hours at room temperature in the dark. After thoroughly washing with PBS three times, a second primary antibody (raised in a different species than the first) was incubated overnight at room temperature in a dark humidified chamber. Finally, a second AlexaFluor-488-conjugated secondary antibody was incubated for 2 hours at room temperature in the dark, and finally, after rinsing in PBS, sections were coverslipped with an aqueous-based mounting media (Vectashield^®^ Hard Set with DAPI; Vector Laboratories), and visualized in an Olympus FV1000 confocal microscope equipped with FluoView software. Confocal scanning of double labeled sides was done with the “Sequential” and Kalman imaging functions of the confocal, which prevents bleaching of the fluorescent signal through different channels and to eliminate background signal, ensuring the accuracy of the images.

### Western Blot for Arginase1 in G-MDSC and CD3ζ Chain in T Cells

Protein extract was obtained by lysing PBMC or purified CD3 cells using Mammalian Protein Extraction Reagent (Thermo Scientific) supplemented with 0.1% SDS and Halt Protease and Phosphatase Inhibitor. Twenty μg (20μg) of protein was loaded into an 8% Bis-tris gel (Life Technologies) for detection of Arg1, or a 4-12% Bis-tris gel for detection of CD3ζ. After transfer to a PVDF membrane it was blocked with 5% milk for 1h and incubated overnight with appropriate primary antibodies. Primary anti-human antibodies included arginase-1 (BD Biosciences), phospho CD3ζ (Abcam), CD3ζ (Abcam), and β-actin. Following incubation HRP was detected using ECL Western Blotting Substrate (Thermo Scientific).

### Gene Expression

Gene expression in PBMC from COVID-19 patients and healthy controls was tested by qRT-PCR using a pre-designed Human Myeloid Derived Suppressor Cell Primer Library from RealTimePrimers.com. Briefly PBMC from healthy controls (n=5), severe (n=6), and convalescent COVID-19 patients (n=6) were centrifuged and RNA was extracted using Qiagen RNeasy Mini extraction kit and used for cDNA synthesis (Thermo Verso cDNA synthesis kit). Amplification by SYBR Green based qRT-PCR reactions were conducted in duplicate and Ct value technical replicates were averaged. ΔCt values of each gene were calculated by normalizing gene expression against the housekeeping control gene PPIA. 2^-ΔΔCt was calculated to determine the gene expression fold change compared to healthy controls, and expressed as the log2 fold change. Error in gene expression was included as the standard deviation of the ΔΔCt. Statistical significance was determined by calculating the p-value using a student’s t-test from the average ΔCt values of either active or convalescent patients compared to control patients. A fold change of an increase of 2 or less than 0.5 was also used as a statistical threshold. Only genes that provided significance in both methods were reported.

### Plasma Arginine

Plasma Arginine was measured high-performance liquid chromatography (HPLC) electrochemical detector (E1 = 150, E2 = 650mV) using a ThermoScientific Dionex Ultimate 3000. Standards of L-arginine were run with each experiment and the levels of the amino acid in a test sample were determined relative to this standard curve. RPMI media (1.15mM L-arginine) was used as an internal control.

### Cytokines, Nitrites, Protein S and PAI-1 Inhibitor

Plasma was used to measure cytokines using a Multiplex assay (Milliplex MAP Human Cytokine/Chemokine Magnetic Bead Panel Millipore-Sigma). Nitrites formed by the spontaneous oxidation of NO were measured by Griess reaction (Molecular Probes). Endothelial cell dysfunction markers were measured by ELISA, namely changes in Protein S (Diapharma Inc. West Chester, OH) and PAI-1 Inhibitor (PAI-1- Abcam, Cambridge, MA).

### RNA Sequencing

RNA sequencing and analysis was done at the Stanley S. Scott Cancer Center’s Translational Genomics Core (TGC; LSUHSC, New Orleans, LA). Briefly, purified G-MDSC were isolated by labeling with anti-CD66b-PE (Clone G10F5; BD Biosciences) followed by positive selection with Human PE Positive Selection kit containing dextran-coated magnetic particles (Stemcell Technologies, Cambridge, MA). The CD66b-PE depleted fraction was used to isolate CD3^+^ T cells by using the T cell negative isolation kit (Stemcell Technologies, Cambridge, MA). Total RNA was extracted from enriched cells by using Qiazol followed by purification with miRNeasy Mini Kit (both from Qiagen, Germany) with an additional DNase I treatment. RNA concentration was determined by fluorometry using the Qubit RNA HS Assay kit (Invitrogen, Thermo Fisher Scientific) and RNA integrity was assessed using the RNA 6000 Pico Kit on an Agilent Bio Analyzer 2100 (Agilent Technologies, Santa Clara, CA). Only samples that showed satisfactory amount and quality were used for RNA-Seq. Paired-end libraries (2 x 75) were prepared (600ng per sample) using the mRNA Stranded Library Preparation Kit from Illumina (Illumina Inc., San Diego, CA). The libraries were validated, normalized and sequenced on a NextSeq 500/550 High Output Kit v2.5 flow cell (Illumina) on the NextSeq500 sequencer (Illumina). FASTQ output files were downloaded from the Illumina BaseSpace and uploaded to Partek Flow. Contaminants (rDNA, tRNA, mtrDNA) were removed using Bowtie2 (version 2.2.5) and the unaligned reads were aligned to STAR (version 2.6.1d) using the hg38 version of the human genome as a reference. Aligned reads were quantified to the hg38-RefSeqTranscript release 93 and normalized by TMM followed by transformation by log2 (1+TMM+log2). Normalized counts were used to determine differential gene expression between patients with or without COVID-19 by using DEseq2 and with a false discovery rate for multiple testing (FDR) of 0.05 (FDR <0.05) and fold change 2 in Partek Flow. Genes with an FDR < 0.05 and fold change (FC) ≥ 2 were considered differentially expressed between the groups. Significantly differentially expressed genes were plotted as heat-maps with hierarchical clustering using the embedded algorithm in Partek Flow for hierarchical unsupervised comparison of the samples using Euclidian distance. The values of expression are visualized with colors ranging from red (high expression) over black (intermediate expression) to blue (low expression). We used MetaCore and Key Pathway Advisor software to predict pathways, networks, gene ontology processes and diseases associated with the differentially expressed genes. Pathways with a positive and negative direction in the Top 25 were selected for further analysis and pathways with no predicted functional consequences were not included. The datasets (FASTQ files, raw counts) are being granted an accession number at the Gene Expression Omnibus (GEO) and will be open to the public upon acceptance of the manuscript.

### Statistical Analysis

Comparisons between groups were done by one-way ANOVA on ranks and Dunn’s Multiple Comparison Test. Two-tailed statistical significance of the Spearman’s coefficient was calculated for correlating variables. Results were considered significant when p ≤ 0.05. All analyses were done using GraphPad Prism 6 software (Graphpad, San Diego, CA).

**Figure 1:**
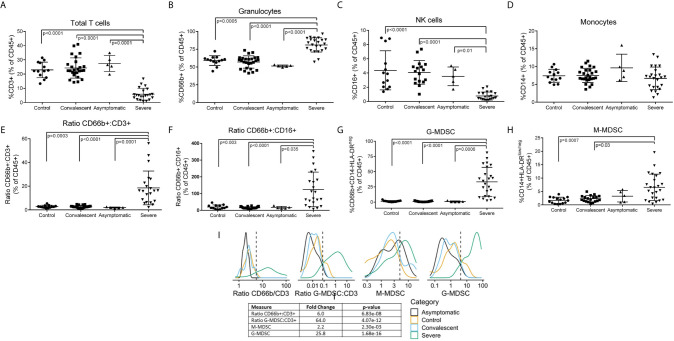
Leukocyte Subpopulations including MDSC in Peripheral Blood of COVID-19 Patients. Whole blood and PBMCs from all participants were analyzed by flow cytometry. Percent **(A)** T cells, **(B)** granulocytes, **(C)** NK cells, and **(D)** monocytes in whole blood. **(E)** CD66b/CD3 and **(F)** CD66b/CD16 ratios in whole blood. Percent **(G)** G-MDSC and **(H)** M-MDSC in PBMC isolated over ficoll-hypaque; Comparisons between groups were done by one-way ANOVA on ranks and Dunn’s Multiple Comparison Test **(I)** Gaussian Kernel Density using a log10-scaled density of observed values for CD66b:CD3 ratios, G-MDSC : CD3 ratios, M-MDSC, G-MDSC. The vertical, dashed line represents the thresholds obtained in the sensitivity/specificity analysis providing the maximum separation between severe COVID-19 patients and other groups of patients or healthy controls.

For further comparisons of the significance and predictive values of the flow cytometry results were calculated using a t-test comparing log-values in the severe group against all other groups combined, without assuming the variances are equal. The log10-scaled observations in **Figure 1I** are smoothed using a Gaussian kernel. Statistics and **Figure 1I** were generated using R 4.0.2 and the following packages: tidyverse (1.3.0) and MESS (0.5.7). To assess the significance of the observed sensitivity, specificity, PPV and NPV, permutation testing was used. In each trial, the ratios or levels of all samples were scrambled and then used to construct a 2x2 contingency table. After 10,000 trials, the number of times the calculated sensitivity, specificity, PPV, NPV, as well as the sum of the sensitivity and specificity, and the sum of the PPV and NPV exceed the observed values were used to determine the 1-tailed probability of observing these values by chance. For example, ratio of CD66b:CD3, After ordering the CD66B:CD3 ratios from largest to smallest, an optimum threshold value was chosen to maximize the values of the sum of the sensitivity plus specificity and the sum of the positive and negative predictive values. This optimum occurred when the threshold was between 5.424 and 5.572. Since the 21 samples with a ratio above the threshold were Severe samples, the specificity and positive predictive value were both 100%. The sensitivity was 87.5% and the negative predictive value was 93.6%.

## Results

### Study Participants

The study was conducted under LSU IRB protocol #20-053 and Ochsner Medical Center IRB protocol 21015-101C. All participants were consented prior to inclusion in the study. Peripheral blood from 24 severe, 26 convalescent and 5 asymptomatic COVID-19 patients and 15 healthy (COVID-19 negative) controls were used for these studies (**Supplemental Table 1**). Severity of the disease was determined following WHO classification. All severe COVID-19 patients were being treated in the ICU at the time of sample collection. Treatments included remdesivir, systemic corticosteroids, antibiotics and anticoagulants; with convalescent sera or monoclonal antibodies used in 3 cases. Only 50% of severe COVID-19 patients were on ventilator support. Samples were collected within the first three weeks of admission to ICU (mean= 17.6 days; range 2-77 days). Asymptomatic and convalescent patients were ambulatory. Average age varied from 43.4 in asymptomatic patients to 67.8 in severe COVID-19 patients. Average Body Mass Index (BMI), a risk factor for severity, ranged from 25 in healthy controls to 32.5 in severe COVID-19 patients. Tissues from 10 patients who died from COVID-19 complications were tested for inflammatory infiltration.

### Dysregulated Immune Profiles in Severe COVID-19

Flow cytometry of peripheral blood (**Figure 1**) showed a significant decrease in CD3+ T cells (**Figure 1A**) (both CD4 and CD8 – data not shown) and CD16+ NK cells (**Figure 1C**), no significant changes in CD14+ monocytes (**Figure 1D**), and a moderate but significant increase in CD66b+ granulocytes (**Figure 1B**). However, CD66b granulocyte: CD3 T cells (**Figure 1E**) and CD16 NK (**Figure 1F**) cell ratios were dramatically increased in severe COVID-19 patients. Separation of peripheral blood mononuclear cells (PBMC) over ficoll-hypaque, which eliminates high density granulocytes, further revealed a significant increase in G-MDSC in severe COVID-19 patients ranging from 2.3% - 76% (mean=29.3%) (**Figure 1G**), which was >10 fold higher than normal controls (mean=2.5%), asymptomatic and convalescent patients (mean=1.2%). In 62% (14/24) of severe COVID-19 patients G-MDSC were >20% of PBMC following ficoll-histopaque separation, i.e. > 4 standard deviations above the mean of normal controls and convalescent patients. G-MDSC levels did not differ between severe COVID-19 patients needing ventilator support, the presence of pneumonia, nor differences in medications (data not shown). Monocytic- MDSC (1H) were also moderately increased, but not as significantly as G-MDSC. Comparison of the flow cytometry results using Log Density Plots (**Figure 1I**) showed significantly higher CD66b/CD3 (p=6.8 x 10^-8^), G-MDSC/CD3 (4.07 x 10^-12^) ratios and percent G-MDSC (1.68 x 10^-16^) in severe COVID-19 patients compared to other patients. These major differences suggest that these ratios and the percent G-MDSC may help identify patients progressing toward a severe form of COVID-19, while suggesting that M-MDSC levels may not be predictive of severe disease.

### G-MDSC Infiltration of the Lungs

**Figure 2:**
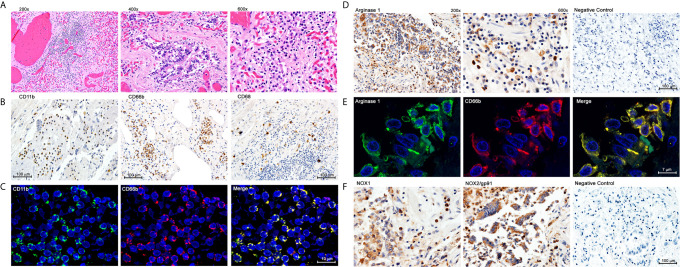
Immunohistochemistry and Double Immunofluoresence of Inflammatory Infiltrates in Lung Autopsy Samples. **(A)** Hematoxylin & Eosin staining. **(B)** Immunohistochemistry with anti-CD11b, CD66b and CD68. **(C)** Double immunofluorescence with anti-CD11b and anti-CD66b. **(D)** Immunohistochemistry for Arginase 1. **(E)** Double immunofluorescence with anti-Arginase 1 and anti-CD66b; **(F)** Immunohistochemistry with anti-NOX1 and anti-NOX2.

A frequent complication of severe COVID-19 is hypoxemia and acute respiratory distress. We tested whether G-MDSC might play a role in the pathophysiology of this complication. Lung autopsy samples from ten patients who died from respiratory distress during severe COVID-19 were tested for inflammatory infiltrates (**Figure 2**). **Figure 2A** shows extensive infiltration of broncho-pulmonary parenchyma by inflammatory cells with extensive damage of alveolar spaces, exfoliation of bronchial epithelium and thrombosis of blood vessels. Immunohistochemistry (**Figure 2B**) showed a prominent expression of myeloid and granulocyte markers CD11b+ and CD66b+, with few CD68+ macrophages and virtually no T cells (data not shown). Double immunofluorescence (**Figure 2C**) confirmed that most inflammatory cells co-expressed CD11b and CD66b, indicating that they are granulocytic cells. Immunohistochemistry confirmed the presence of large numbers of Arg1^+^ cells (**Figure 2D**). Double immunofluorescence (**Figure 2E**) demonstrated a high expression of granular intracytoplasmic Arg1 in CD66b+ confirming them to be Arg^+^G-MDSC. Furthermore, staining with anti-NOX-1 and NOX-2 antibodies revealed high expression of these enzymes in clusters of inflammatory and exfoliated epithelial cells (**Figure 2F**). NOX-1 and NOX-2 enzymes catalyze the synthesis of reactive oxygen species (ROS). These results suggest that the massive infiltration of the lungs by Arg^+^G-MDSC simultaneously expressing NOX1/2, may inhibit T cells and cause endothelial cell dysfunction (through depletion of arginine, which is required for nitric oxide production and blood vessel tone), and directly damage alveolar epithelium through the release of ROS. This may in part explain the acute respiratory distress syndrome and coagulopathy frequently seen in severe COVID-19 patients. The high numbers of Arg^+^G-MDSC in circulation and lungs of COVID-19 patients, contrasts with the findings in cancer where G-MDSC concentrate around tumors with increases in peripheral blood found mostly in patients with advanced disease (19–21).

### Metabolic and Functional Consequences of Increased Arg^+^G-MDSC

**Figure 3:**
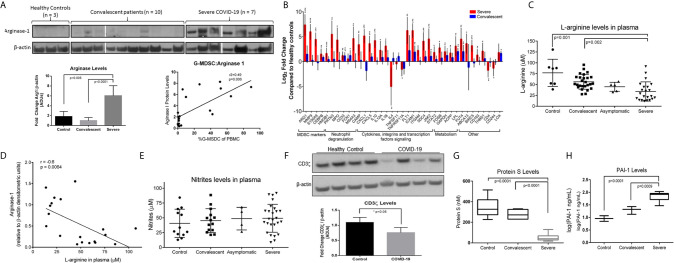
Effects of Increased Arg^+^G-MDSC. **(A)** Arginase-1 protein expression in PBMC from severe, convalescent COVID-19 patients and healthy controls with associated quantification of fold-changes. **(B)** Gene expression comparison by SYBR green qRT-PCR of PBMC from severe (red bars) and convalescent (blue bars) and healthy controls (baseline). X axis shows Log2 fold change in gene expression. Asterisks denote *p ≤ 0.05, **p ≤ 0.01, ***p ≤ 0.001, and ****p ≤ 0.0001, using Student’s t-test from the average ΔCt values. **(C)** L-arginine concentration in plasma of patients and healthy controls **(D)** Correlation of Arg-1 protein expression in PBMC and plasma arginine. **(E)** Plasma nitrate levels in plasma. **(F)** CD3ζ chain expression in purified T cells from 4 representative healthy controls and 4 COVID-19 patients with average densitometry shown below with a student’s t-test with Welch’s correction. **(G)** Plasma protein S and **(H)** plasminogen activator inhibitor-1 (PAI-1) in plasma.

**Figure 4:**
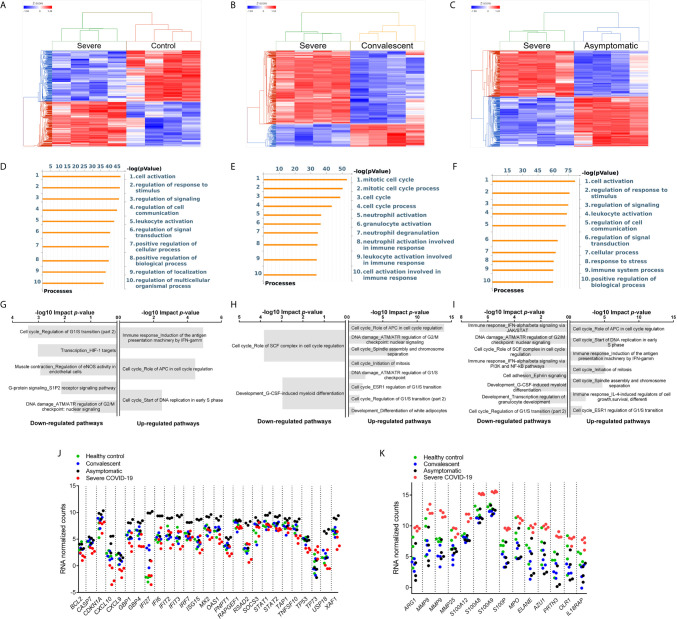
Differential Gene Expression in G-MDSC from Patients and Healthy Controls. Comparison of the transcriptome of purified G-MDSC using RNAseq from severe (n=4), asymptomatic (n=4), and convalescent (n=4) COVID-19 patients and healthy controls (n=4). **(A–C)** Dendrograms and Heat-Maps for Unsupervised Hierarchical Clustering comparing transcriptome expression. **(D–F)** Differences in Gene Ontology Processes identified by *MetaCore* in G-MDSC from patients and healthy controls. **(G–I)** Analysis using Key Pathway Advisor software identified the Top 25 differentially expressed pathways. **(J, K)** Dot plots comparing differentially expressed genes of “Immune response IFNalpha/beta signaling *via* JAK/STAT” **(J)** and Granulocyte Functions and Degranulation **(K)** in G-MDSC from patients and healthy controls.

G-MDSC can deplete arginine and impair T cell and endothelial cell function (18, 22, 23). **Figure 3A** shows representative Western blot data from 7 severe and 10 convalescent COVID-19 patients and 3 healthy controls. All severe COVID-19 patients had high Arg1 protein expression, compared to only 2/7 convalescent patients tested and none of the healthy controls. Quantification of these Arg1 expression levels reveals a roughly 5-fold increase in Arg1 in severe COVID-19 patients compared to controls and convalescent individuals. There was also a positive correlation between the number of G-MDSC and ArgI expression in these samples. SYBR Green qRT-PCR using an MDSC primer panel confirmed a 7.5 fold higher expression of Arg1 in the PBMC of severe COVID-19 patients compared to convalescent and healthy controls (**Figure 3B**). These results also showed increased expression of genes associated with MDSC including MMP9, S100A9, CEBP and genes associated with granulocyte functions such as myeloperoxidase (MPO) and neutrophil degranulation proteins PRTN3. These findings were further confirmed by RNAseq from purified G-MDSC (shown in **Figure 4**)

The metabolic consequence of high numbers of Arg^+^G-MDSC was a significant decrease in plasma arginine in severe COVID-19 patients (mean=45μM; range 18-120μM), compared to healthy controls (mean=75μM; range 50-130μM) and convalescent patients (mean=55μM; range 26-92) (**Figure 3C**). As expected, there was an inverse correlation between Arg1 expression (by W. blot) and arginine plasma levels (**Figure 3D**), but nitrite levels were unchanged (**Figure 3E**). These observations are significant in that T cells cultured in arginine <50μM lose the T cell receptor zeta chain (CD3ζ) expression, impairing proliferation and IFNγ production (15, 22). This effect was also observed in purified T cells from severe COVID-19 patients tested (**Figure 3F**), which showed more than 25% reduction in CD3ζ expression. Arginine depletion also causes endothelial cell dysfunction through interfering with nitric oxide production, which is required for maintenance of blood vessel tone (18). This was confirmed here by decreased levels of protein S (**Figure 3G**) and increased plasminogen activator inhibitor-1 (PAI-1) (**Figure 3H**), which can also increase the risk for intravascular coagulation.

### G-MDSC from COVID-19 Patients Have Stage-Specific RNA Expression Profiles

To better understand the differences in inflammatory mechanisms in patients with different stages and severity of COVID-19, we compared the transcriptome of purified G-MDSC using RNAseq. Unsupervised hierarchical cluster analysis of the differentially expressed genes (DEGs) in G-MDSC from severe COVID-19 patients *vs* healthy controls (**Figure 4A**), convalescent (**Figure 4B**) and asymptomatic patients (**Figure 4C**) demonstrated clear differences between these groups. A Venn diagram (**Supplemental Figure 1**) illustrating shared and unique genes revealed the most significant differences in RNA transcripts (FDR <0.05; fold change ≥ 2) was between severe and asymptomatic patients (3675 transcripts), followed by severe and healthy controls (2452 RNA transcripts) and severe and convalescent (863 genes). An initial analysis using *MetaCore* software identified gene ontology processes that were significantly different among the groups. Specifically, G-MDSC from severe *vs* convalescent patients showed major differences in the expression of genes associated granulocyte functions and degranulation (**Figure 4E**). In contrast, differences between severe COVID-19 patients and healthy controls or asymptomatic patients were primarily associated with normal cell activation, signaling and regulation (**Figures 4D, E**). Additional analysis using *Key Pathway Advisor* software identified the top 25 Pathways with predictive positive or negative functional consequences. Comparison of severe *vs* asymptomatic patients showed that G-MDSC from severe patients had significantly decreased expression of genes from pathways associated with type I IFN responses (Immune response_IFN-alpha/beta signaling *via* JAK/STAT) and its down-stream signaling mechanisms (Immune response_IFN alpha/beta signaling *via* PI3K and NF-κB pathways) (**Figure 4I**). To further illustrate these findings we plotted the expression of representative genes associated with granulocyte functions and genes associated with type I IFN pathways. These data confirmed increased expression of genes associated with anti-viral responses such as IFI27, IFIT2 and IFIT3 (24–27) in G-MDSC from asymptomatic patients (**Figure 4J**). In contrast, G-MDSC from severe COVID-19 patients had increased expression of genes associated with granulocyte functions/degranulation and chronic inflammation including Arg1, MMP8, MMP9, S100A8 and A9, MPO, IL18RA, elastase, PRTN3 (azurophilic granule protein 7) (**Figure 4K**). These results demonstrate contrasting inflammatory and immune responses between asymptomatic and severe COVID-19 patients. The increased expression of type I IFN associated pathways and down-stream signaling mechanisms in G-MDSC from asymptomatic patients suggested the development of a protective anti-viral immune response. It is unclear whether G-MDSC can themselves have anti-viral activity or serve as effective antigen presenting cells. In contrast, patients with severe COVID-19 develop a granulocyte inflammatory response that exacerbates the disease. What genomic characteristics regulate the degree and type of inflammatory response is still unclear, but these findings may help identify individuals that are more likely to develop severe disease. The complete lists of differentially expressed genes are included in **Supplemental Table 2**.

## Discussion

These data demonstrate the importance of Arg^+^G-MDSC in the pathophysiology of severe COVID-19. As previously reported arginine depletion impairs T cell and endothelial cell function, and can be expressed by multiple cell types, including MDSCs and macrophages. This study primarily found that G-MDSCs were increased in both the peripheral blood and lung tissue of severe COVID-19 patients; however, macrophages were also present to a lesser degree. The levels of G-MDSC did not correlate with other demographic parameters such as age, BMI, or race. While we did not directly test for M-MDSC in the lung tissue, other studies provide evidence that they are not elevated in lungs of severe COVID-19 patients, while they are increased in peripheral blood (8). Together, we believe these findings point to a prominent role for Arg+G-MDSC in the pathology of COVID-19 in the lungs. Here we further demonstrate infiltration of the lungs by Arg^+^G-MDSC expressing NOX2, which possibly release ROS causing direct damage of the pulmonary epithelium and increased inflammation, resulting in acute respiratory distress. Why G-MDSC show a preference for bronchoalveolar tissues and not other organs (data not shown) is unclear. It is possible that release of damage associated molecular patterns (DAMPs) from infected respiratory epithelium activate G-MDSC. Similar observations have been made in patients with COPD where DAMPs increase inflammation and fibrosis (28, 29). Alternatively, lipopolysaccharides (LPS) from bacterial pneumonia, which frequently accompanies severe COVID-19, could exacerbate the response by G-MDSC. Severe alveolar damage caused by inflammatory cytokines and ROS has been well documented as a mechanism for pathogenesis of acute lung injury and adult respiratory distress syndrome (30). Patients with Severe COVID-19 studied here also had significantly increased levels of inflammatory cytokines in plasma (**Supplemental Figure 2**).

Arginine depletion can also be a mechanism for endothelial cell dysfunction and increased intravascular coagulation. Inhibition of arginase-1 restores endothelial function and production of nitric oxide in studies of cardiovascular disease (16, 17, 31, 32). Our data shows that severe COVID-19 patients had decreased plasma levels of Protein S, suggesting it is rapidly being consumed, and increased PAI-1, which is a direct manifestation of endothelial cell dysfunction. Arginase can also decrease NO production by endothelial cells because of its faster kinetics and higher avidity for arginine compared to nitric oxide synthase 2 (NOS2). Thus, arginine deficiency can cause vasoconstriction, increase platelet adherence, and further promote hypercoagulation (32).

Another well-established property of MDSC is their ability to inhibit T cell responses in multiple inflammatory conditions including cancer, tissue trauma, and chronic infections. MDSC contribute to the establishment and maintenance of infections by producing multiple factors that inhibit T cell responses (33). Perhaps primary among these are the production of Arg1, which inhibits T cells in multiple ways. The first is through inhibition of T cell expansion, due to the reliance of T cells on arginine to proliferate (34). The second mechanism is through the inhibition of signaling through the T cell receptor zeta chain (35). Together these two result in fewer T cells that are unable to function properly, which can lead to reduced anti-viral or anti-microbial responses and prolong or amplify infections. While we did not directly measure G-MDSC effects on T cell proliferation, we did observe reduced T cells in the severe COVID-19 patients, as well as a reduced expression of CD3ζ in a subset of these patients that were tested. These two findings suggest that greatly increased G-MDSC numbers in the severe COVID-19 patients contributed to the reduced T cell number and reduced signaling through the CD3ζ chain. We believe together that these implicate Arg+G-MDSC in the progression to severe disease.

Previous RNA-seq studies from COVID-19 patients have shown dysregulated immune profiles (36, 37). A recent study by Combes et al. showed a predominance of interferon-stimulated genes (ISG – including IFIT1-3) in all immune cells tested (neutrophils, macrophages, T and NK cells) from patients with mild COVID-19 (38). Some of the ISG genes identified in G-MDSC in our study were also increased in one of seven neutrophil subsets studied by these investigators. ISG expression declined in patients with severe disease. Therefore, both studies confirm the increased expression of ISG in asymptomatic (mild) COVID-19 patients compared to their expression in severe COVID-19 patients, which further strengthens the concept that ISG pathways are critical for a protective immune response to SARS-Cov-2. In addition to these alterations in ISGs, both our data and theirs reveal an increase in markers that are associated with G-MDSC activation, including Arg1 and S100A12, indicating that activated Arg1+G-MDSC play a role in severe COVID-19.

These observations also suggest novel therapeutic approaches including the use of arginase inhibitors or the replenishment of arginine. Arg1 inhibitors are currently in early phase clinical trials in cancer (Calithera/Incyte (39, 40)), while the replenishment of arginine or arginine precursors has been tested in sickle cell disease resulting in a significant reduction in vaso-occlusive complications (41, 42). Arginine replenishment has also been tested in surgery where it successfully blunted the surge of G-MDSC, prevented T cell dysfunction and decreased infectious complications (43–45). Therefore arginase 1 inhibition and/or arginine replenishment should be considered as an adjuvant to the prevention/treatment of COVID-19.

## Data Availability Statement

The data presented in the study are deposited in the Gene Expression Omnibus repository under accession number GSE178824 (https://www.ncbi.nlm.nih.gov/geo/query/acc.cgi?acc=GSE178824).

## Ethics Statement

The studies involving human participants were reviewed and approved by LSU Health Sciences Center Institutional Review Board and Ochsner Medical Center Institutional Review Board. The patients/participants provided their written informed consent to participate in this study.

## Author Contributions

MJD, MDS-P, RT, DW, PP: Processed samples, conducted flow cytometry, W blots, HPLC and qPCR. LDV, LD, RVH: Immunohistology. JZ, JG, CH, JW: RNA seq - transcriptome and analysis. JBO, JGD, LB, BN, WMR: Identified and consented patients, collected samples and clinical data. RM: Endothelial dysfunctionAC, JC, BL, RJ: Statistical analysis. CAR, CRM, HK: Provided critical input on data interpretation, new assays and manuscript development. ACO: Overall planning and execution, data interpretation, manuscript preparation. All authors contributed to the article and approved the submitted version.

## Funding

This project was supported by funding from LSU Health and Ochsner Medical Center. Core facilities, personnel and services partially supported by an internal LSU Health COVID-19 grant as well as P20-GM103501, P20-CA233374, P20-GM121288, and U54-GM104940. This project has been funded in part with Federal funds from the National Cancer Institute, National Institutes of Health, under Contract No. HHSN261200800001E. The content of this publication does not necessarily reflect the views or policies of the Department of Health and Human Services, nor does mention of trade names, commercial products, or organizations imply endorsement by the U.S. Government.

## Conflict of Interest

The authors declare that the research was conducted in the absence of any commercial or financial relationships that could be construed as a potential conflict of interest.
